# Childhood habit cough treated with consultation by telephone: a case report

**DOI:** 10.1186/1745-9974-5-2

**Published:** 2009-01-21

**Authors:** Ran D Anbar

**Affiliations:** 1 Department of Pediatrics, State University of New York Upstate Medical University, Syracuse, NY, USA

## Abstract

**Background:**

Childhood habit cough has been treated successfully by making suggestions that it can be stopped, desensitization techniques, use of distractors, provision of rewards, and self-hypnosis. All of these techniques have involved personal contact between a health care provider and a patient.

**Case presentation:**

A 5-year-old with cystic fibrosis was diagnosed with habit cough following evaluation by a pediatric pulmonologist and otolaryngologist. An expert in the treatment of habit cough provided instruction by telephone to the patient's mother regarding use of hypnotic techniques in this setting, which was associated with resolution of the cough within a week.

**Conclusion:**

As this report describes a single patient, it is possible that his improvement was unrelated to the given advice. Therefore, it remains to be seen whether therapy by telephone for habit cough is applicable widely.

## Background

Childhood habit cough has been treated successfully by making suggestions that it can be stopped, desensitization techniques, use of distractors, provision of rewards, and self-hypnosis [1]. All of these published techniques have involved personal contact between a health care provider and a patient.

This report presents a child with cystic fibrosis (CF) who was diagnosed with habit cough. After consultation by telephone with a pediatric pulmonologist, the cough improved rapidly following employment of suggested hypnotic techniques.

## Case presentation

The patient was an almost 5-year-old, diagnosed with CF following birth with meconium ileus. He was homozygous for the ΔF508 CF mutation. He developed pneumonia at 3 months, and by a year of age was found to harbor *Pseudomonas aeruginosa *in his airway. Since then he was treated with every-other-month tobramycin by inhalation (TOBI^®^), and daily Pulmozyme.

Four months before he developed habit cough, the patient manifested severe coughing after contracting influenza. At that time, he was diagnosed with a second pneumonia, and treated with intravenous antibiotics. Two months later, computerized tomography revealed diffuse sinusitis, which was thought to be aggravating his pulmonary disease. Therefore, he underwent sinus surgery. A week after removal of the post-surgical nasal packing he developed a loud, harsh cough that occurred every other second for hours at a time, while awake. The cough ceased as soon as he fell asleep.

The patient was treated with oral ciprofloxacin and prednisolone in addition to TOBI^®^, but there was no improvement in his cough. No new abnormalities were documented on his physical examination. His forced expiratory volume in one second was 145% of predicted for his height and age. His pediatric pulmonologist and otolaryngologist concurred that he likely had developed habit cough. The family was reassured that the cough would resolve in time. In order to help the cough resolve, it was suggested the patient be told, "Are you sure you need to be coughing?" However, the mother felt ambivalent about this advice because she was aware that coughing is beneficial for patients with CF. No further therapy was offered. Because of the cough the patient was unable to attend summer camp.

The patient lives with his parents, and 6-year-old sister who also has CF. The sister's respiratory status is good, but she has a gastrostomy tube for supplemental feedings.

The patient is scheduled to be enrolled in kindergarten next year. He enjoys playing with friends, toy cars, and swimming.

As the cough persisted for a month, his mother decided to research habit cough on the internet, and found an article about its treatment with self-hypnosis [1]. She called the pediatric pulmonologist author of this article and asked whether he would be willing to evaluate the child. She wanted to fly with the patient to meet the physician, as the family lives 1500 miles away.

During a 15 minute telephone conversation, the physician suggested that before deciding whether to make the trip, the family could use some hypnotic techniques on their own. These included: (1) Telling the patient, "When you get better it will be great" to participate in a fun activity. (2) Asking the patient, "What would you like to do once you get better?" (3) Suggesting that as he does not cough when he swims, if he would like not to make a noise, he might imagine swimming. (4) Finding a sea shell with magical anti-noise properties during a visit to the sea shore.

The family employed all four of the suggestions. Additionally, following the telephone discussion, his mother realized it would be safe to tell the patient to stop his habit "noise", since it was not the same as his CF cough. Within a week, the patient's cough decreased in frequency to approximately once hourly. His mother wondered whether the residual cough was related to his CF lung disease.

## Discussion

Habit cough typically occurs in association with respiratory illness [1], although a review of the literature did not reveal a previous report of its occurrence in a patient with CF. Psychosocial stressors frequently are associated with habit cough [1], and in the case of the described patient could have included the burden of his CF, including his recent pneumonia and surgery, and his sister's CF.

Prior to use of therapy by telephone, it is imperative to ensure that patients have undergone a comprehensive medical evaluation in order that any associated physiological abnormalities can be diagnosed and treated. In the reported case, such evaluation was performed by a pediatric pulmonologist and an otolaryngologist before the suggestions were made by telephone. Further, resolution of the cough following the behavioral intervention is consistent with a diagnosis of habit cough [2].

Each of the techniques recommended to the patient's mother had hypnotic features [3], which were based on the patient's developmental age and interests. (1) "When" (rather than "if") you get better" carries an implicit suggestion that the cough will resolve. (2) Visualization of a healthy future promotes improvement, and was prompted by querying about his plans once he is well. (3) Imagery can help resolve habit cough [1], which was encouraged by the suggestion regarding imagined swimming. (4) The magic sea shell may have helped by utilizing this young patient's belief in its effectiveness, and/or served as a physical reminder that the cough can be controlled [1].

The success of the advice also may have been related to young children's recognized responsiveness to suggestion [4], and the perceived authority and knowledge of the physician who was an expert in treatment of both habit cough and CF. Further, alleviation of the family's anxiety regarding the safety of suggestions regarding the cough may have been an important factor.

Sometimes, habit cough does not resolve until underlying psychosocial stressors are addressed extensively [1,5]. In such situations, simple hypnotic suggestions likely are insufficient, and therefore advice given by telephone is unlikely to work. Further, older patients may be more likely to require a personal meeting with a physician in order to allow establishment of rapport that typically serves as the foundation for a successful hypnosis intervention [5,6]. Thus, if a presumed habit cough fails to resolve, patients should be referred for a personal evaluation by an expert in the treatment of this condition.

## Conclusion

As this report describes a single patient, it is possible that his improvement was unrelated to the given advice. Therefore, it remains to be seen whether therapy by telephone for habit cough is applicable widely. If such therapy is effective, its financial consequences will need to be considered, as insurance companies typically do not reimburse physicians for intervention by telephone. An alternative could be interactive video teleconferencing that currently is reimbursed and allows provision of effective mental health therapy, which has been well received by children and their parents [6].

## Consent

Permission was obtained from the patient's mother for publication of this report.

## Competing interests

The author declares that they have no competing interests.

## Authors' contributions

RA is the physician who treated the patient in this report, and wrote the manuscript.
